# The Effect of Cellulose Nanocrystal-Based Nanofluid on Milling Performance: An Investigation of Dillimax 690T

**DOI:** 10.3390/polym15234521

**Published:** 2023-11-24

**Authors:** Üsame Ali Usca

**Affiliations:** Department of Mechanical Engineering, Bingöl University, 12000 Bingöl, Türkiye; ausca@bingol.edu.tr

**Keywords:** cellulose nanocrystals nanofluid, Dillimax 690T machinability, sustainability C/L conditions, advanced milling

## Abstract

Machining high-strength structural steels often requires challenging processes. It is essential to improve the machinability of such materials, which are frequently needed in industrial manufacturing areas. Recently, it has become necessary to enhance the machinability of such materials using different nanopowders. In this study, different cooling/lubricating (C/L) liquids were prepared with cellulose nanocrystal (CNC) nanopowder. The aim was to improve the machinability properties of Dillimax 690T material with the prepared CNC-based cutting fluids. CNC nanopowders were added to 0.5% distilled water by volume, and a new nanofluid was produced. Unlike previous studies, base synthetic oil and CNC-based cutting fluid were sprayed on the cutting area with a double minimum quantity lubrication (MQL) system. Machinability tests were carried out by milling. Two different cutting speeds (Vc = 120–150 m/min), two different feed rates (f = 0.05–0.075 mm/tooth), and four different C/L environments (dry, MQL oil, CNC nanofluid, MQL oil + CNC nanofluid) were used in the experiments. In the study, where a total of 16 experiments were performed, cutting temperature (Tc), surface roughness (Ra), tool wear (Vb), and energy consumption results were analyzed in detail. According to the test results, significant improvements were achieved in the machinability properties of the material in the experiments carried out using CNC nanofluid. In particular, the hybrid C/L environment using MQL oil + CNC nanofluid improved all machinability metrics by over 15% compared to dry machining. In short, using CNC nanopowders offers a good milling process of Dillimax 690T material with effective lubrication and cooling ability.

## 1. Introduction

Dillimax 690T (Strenx 700 E or S690QL) structural steel has high yield strength and excellent toughness properties due to its high strength and low alloy [1,2]. This industrial material type has attracted significant attention in recent years due to its superiority in working conditions, even in the most challenging environments [3]. There is very little research on the machinability of these materials in the literature. Aslan et al. [4] investigated the effects of dry and MQL environments on the machining and sustainability index of Strenx 900 steel. Gitanjali et al. [5] reported the surface roughness values to measure the machinability properties of Strenx 900 steel via the electrochemical grinding process. Raguraman et al. [6] examined the machinability properties of Strenx 700 material in water jet machining. Although these materials have widespread use, they are considered difficult to machine [7]. This can be attributed to the low thermal conductivity of these materials [8].

The CNC machine uses electrical energy and converts it into mechanical energy to perform the milling process. During the cutting process, this energy mostly turns into heat energy [9]. Although up to 80% of the heat discharge during milling is due to chip discharge, residual heat significantly affects the cutting tool and workpiece (w/p) [10]. Thus, cutting fluid or coolant is used to control residual heat. Preventing excessive heat generation using the correct cooling or cutting fluid is one of the most effective methods. Dry machining has become a choice for researchers and industry because it is sustainable and inexpensive compared to using cutting fluids [11]. However, the absence of a cooling/lubricating (C/L) environment in dry machining causes high cutting temperatures in the cutting processes. For this reason, rapid wearing of cutting tools causes poor surface quality and extra energy consumption [12]. Despite having similar drawbacks as those of dry machining, mineral, synthetic, and semi-synthetic oils were used in flood C/L environments [13]. Thus, better machinability metric results were obtained versus dry machining [14]. As a requirement of developing technology and sustainable manufacturing, MQL systems that consume less cutting fluid have attracted the attention of researchers [15]. In the MQL system, the liquid is sprayed in minimal amounts in a pulverized manner between the cutting tool and the w/p. Thus, MQL fluid forms a thin film where it is located, significantly reducing the friction forces and coefficient. It is known that using MQL effectively increases cutting tool life and w/p surface quality [16]. Ji et al. [17] used MQL to drill Ti6Al4V alloy. They reported that the MQL technique reduced energy consumption and improved w/p surface quality. The advantages of using the MQL technique in the machinability of materials have been stated in many studies in the literature [18,19,20,21,22]. However, the desire to obtain better C/L conditions and the fact that powder metallurgy is present in every field have led to cutting fluids to which nanoparticles are added. Nanofluids are formed by dissolving nanoparticles in base liquids such as water, glycol ethylene, vegetable oil, and synthetic oil [12].

Nanofluids have recently attracted the attention of researchers for increasing tool life, providing better cutting, and obtaining better surface quality. In addition to reducing the total production cost, these fluids also attract attention regarding energy consumption and carbon footprint. Babu et al. [23] examined the machinability of graphene-based nanofluid in Hastelloy C276 compared with other C/L environments (dry, MQL). They reported that graphene-based nanofluid gave the best results for all output parameters. Yücel et al. [24] carried out a study on MoS_2_-based nanofluid. They said it significantly improved surface roughness and cutting temperature compared to MQL. In a study, they prepared hybrid nanofluids using hexagonal boron nitride (hBN), graphite, and MoS_2_ nanoparticles and examined their effects on machinability. They stated that hBN/graphite hybrid nanofluid showed superiority in all machinability criteria compared to graphite/MoS_2_ and hBN/graphite nanofluids [25]. It was reported in the literature that many nanoparticles (Al, Cu, Ni, Al_2_O_3_, TiO_2_, SiO_2_, CuO, and graphene) can be used in nanofluid [12,26]. The fact that nanofluids are not used industrially brings some questions to mind. Therefore, the recycling and reuse of the base liquids and nanopowders used, their effects on human health, and their impact on the environment are not fully known. Nowadays, the need for sustainable manufacturing and environmentally friendly nanopowders and nanofluids obtained from them with minimized adverse effects on human health for processable C/L environments continues to increase.

Cellulose is a crucial polysaccharide and one of the abundant organic polymers in nature [27]. With its many superior physical and chemical properties, this structure also has biocompatibility, biodegradability, and renewability properties [28]. Therefore, CNC-based nanoparticles have attracted the attention of researchers in recent years [29]. However, although CNC has excellent potential for use, its use as a nanofluid in machining is almost non-existent and is still waiting to be discovered. This is also encouraged [30]. In this context, Şap et al. [31] examined the contribution of different CNC-based nanofluid concentrations to the machinability properties in milling polymer materials. They looked at surface roughness, tool wear, and cutting temperature, which are machinability metrics, and emphasized that CNC-based nanofluid significantly contributed to these metrics’ development. They did not analyze energy consumption, which is one of the sustainability parameters. In addition, the effect of these nanofluids on the machinable metal is still unknown.

High-strength or structural steel use continues to increase in various industrial areas. Therefore, improving the machinability of these materials has become a necessity. To achieve this development, researchers continue to produce new nanofluid C/L environments using different nanopowders. However, today, the desire to ensure that growth continues in a sustainable, environmentally friendly, and harmless way to human health restricts using unsustainable nanofluids in the industrial field. This is a significant problem for nanofluids. This study discusses CNC nanofluid obtained from CNC nanopowders, a new subject in literature studies, whose effect is still unclear for structural steels with difficult machinability. Hybrid (MQL oil + CNC-based nanofluid) C/L environments were obtained with CNC-based nanofluid. Among the sustainable C/L environments in production processes, water-based CNC nanofluid was applied for different cutting parameters (Vc = 120–150 m/min and f = 0.05–0.075 mm/tooth). It was compared with different C/L environments (dry, MQL) to monitor the effect of the new nanofluid produced. A comparison was made with other liquids to see the impact of CNC nanoparticles. To evaluate the machinability of Dillimax 690T material, cutting temperature, w/p surface roughness, tool wear, and energy consumption parameters were considered.

## 2. Materials and Methods

### 2.1. Workpiece Material and Cutting Tools Details

This machinability study used the Dillimax 690T material (Birçelik, İstanbul, Türkiye) which has high strength and excellent toughness properties. The materials were supplied in dimensions of 50 × 50 × 25 mm. A new surface of the material was used for each experiment. The chemical properties of the material are given in Table 1.

ISCAR’s Al-TiN-coated HM 90 APKT 1003PDR IC908 (Iscar, Konya, Türkiye) insert was used in the experiments. The insert length is 11.45 mm, the insert width is 6.76 mm, the insert thickness is 3.53 mm, and the corner radius is 0.8 mm. The insert was installed on a single tooth milling cutter with code ST90 AP10 D12 W12 L120 Z01 (Smoxh, Konya, Türkiye). The milling cutter machine was connected to the MAS 403 BT 40 tool holder with a clamping length of 40 mm. One insert was used for each experiment, and the inserts were renewed for each experiment.

### 2.2. Machine Tool and Experiments Procedure

Machinability experiments by milling were carried out on a 3-axis Dahlih MCV-860 horizontal machining center (Dahlih, Taichung, Taiwan) with a maximum rotation speed of 10,000 rpm and a power of 7.5 kW. The compressor was common to the devices used in the experiments. Sixteen experiments were carried out according to the full experimental design system at different cutting speeds of 120–150 m/min, two feed rates of 0.05–0.075 mm/tooth, and four different C/L environments (Table 2).

**Figure 1:**
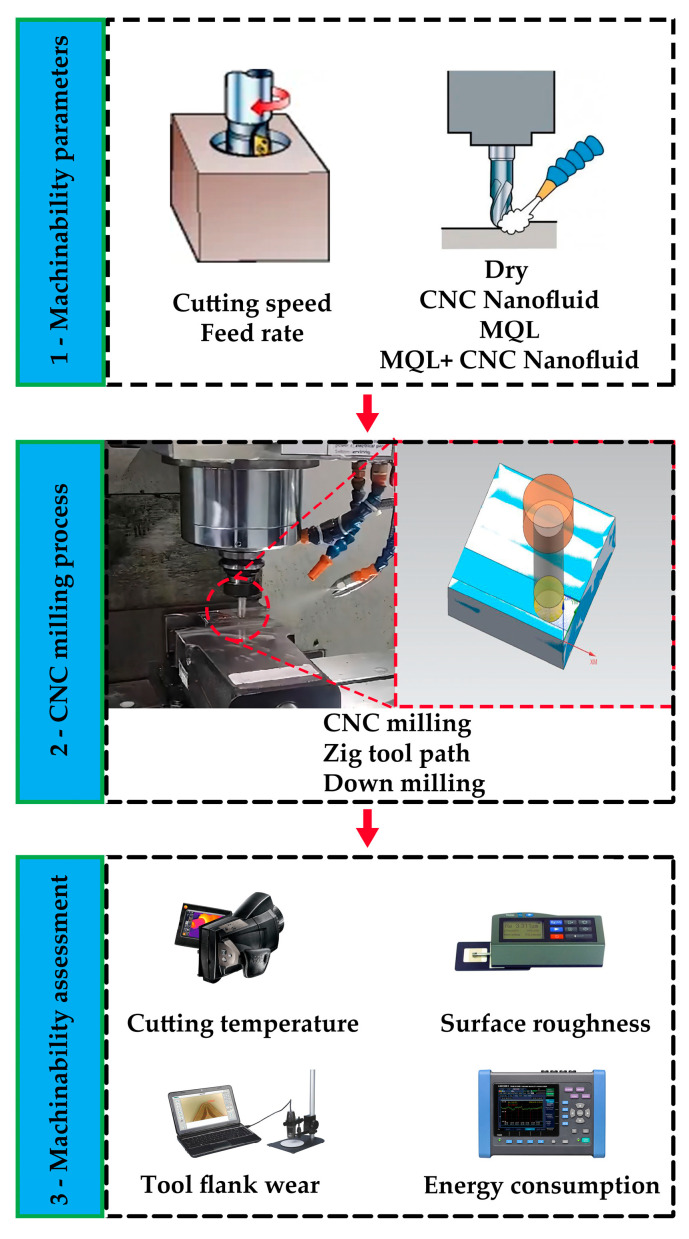
Experimental setup used in milling experiments.

The parameters recommended by the cutting tool manufacturer were effective in the selection of cutting parameters. Before each experiment, the thin layer on the materials was cleaned. During the experiment, the cutting depth was kept constant at 1 mm and the cutting width was kept constant at 12 mm. The milling process used the “Zig” tool path and the down milling (climb milling) strategy. Figure 1 represents the experimental scheme performed.

### 2.3. Cooling/Lubricating Conditions

Four different C/L environments (dry, MQL oil, CNC-based nanofluid, and MQL oil + CNC-based nanofluid) were preferred for the study. Dry milling, which is the process of performing the milling process without using any coolant, was chosen because it is within the scope of sustainable processing [32]. For the MQL C/L condition requirement, a Werte Micro STN-15 MQL (Kar-Tes, İstanbul, Türkiye) system was used. The system working pressure was set as 7 bar. Additionally, a nozzle with an inner diameter of 3 mm was used in the system. This nozzle was kept fixed at a distance of approximately 175 mm from the cutting zone. The fluid flow rate of the system was determined as 40 mL/h. The MQL system was used for three different environments. In the experiments carried out with MQL lubrication only, KT 2000 synthetic oil, which has high hydrodynamic lubrication properties and can be used in spraying systems, was preferred. The most important priority of the study is to observe how cutting fluids prepared with CNC nanopowders affect the machinability of w/p compared to other C/L environments. CNC nanopowders were obtained from the manufacturer (Nanografi, Ankara, Türkiye). Crystalline Nanocellulose is composed of nano-sized cellulose fibrils. Technical properties of CNC nanopowders are given in Table 3.

**Figure 2:**
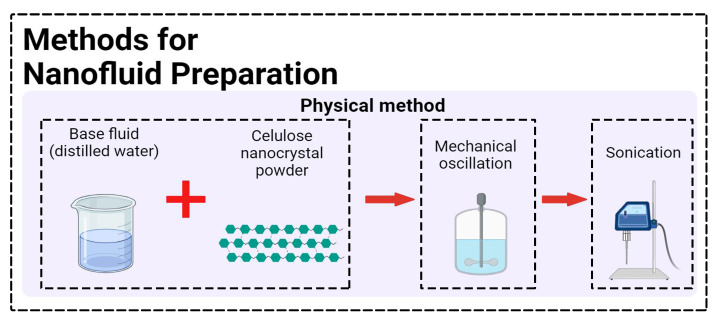
CNC nanofluid hazırlanmasının şematik gösterimi.

CNC nanopowders are soluble in water, so distilled water was used as the base fluid for the nanofluid. According to previous experience [31], CNC nanopowder powders were added to distilled water at 0.5% by volume. A quantity of 500 mL nanofluid was prepared for each cutting fluid. To dissolve the nanopowders by dispersing them into the base liquid, they were mixed with a mechanical stirrer (30 s) and then stabilized with an ultrasonic sonication (1 h). Figure 2 represents the template for the preparation of nanofluid.

Finally, a hybrid (MQL oil + CNC nanofluid) cooling system was applied in the experiments. Since the nanofluids prepared with the synthetic oil (Werte-KT 2000) used for the MQL system do not dissolve in each other, they were sprayed into the cutting zone using MQL systems with the same features.

### 2.4. Measuring Equipment Details

To examine the effect of different machinability parameters on the experimental results, surface roughness, cutting temperature, cutting tool flank wear, and power consumed were measured (Figure 1).

A Time 3200 (Time, Beijing, China) surface roughness measuring device was used for surface roughness measurements. Measurements were made seven times in different regions of each experimental sample. Average surface roughness values (Ra) were determined by subtracting the largest and smallest values and averaging the remaining values.

The cutting temperature (Tc, °C) between the cutting tool and workpiece was determined with a Testo 885 thermal camera (Testo, İstanbul, Türkiye). The images were obtained during the final machining process on the workpiece before the cutting tool finished the milling process. The thermal camera distance to the cutting zone was approximately 500 mm.

The amount of flank wear (Vb_max_) occurring in the flank region of the cutting tool was determined with the Insize ISM PM200SB (Insize, Suzhou New District, China) digital measurement camera. In addition, scanning electron microscope (SEM) and energy dispersive X-ray (EDX) images were obtained to detect wear mechanisms in the cutting tool. Additionally, mapping analysis for cutting tools was also carried out. These analyses were carried out on the JEOL JSM 6510 SEM device (Jeol, Tokya, Japan).

Finally, power measurements were made on the HIOKI PW 3198 power analyzer (Hioki, Nagano, Japan) device during the experiment. The device measures current–voltage values from three phases and produces the results as active power. These results were edited with device-specific software, and the energy consumed was calculated.

## 3. Results and Discussion

In this section, the cutting temperature, surface roughness, flank wear, and power consumed results obtained from the computer numerical control milling process of Dillimax 690T materials are given and discussed.

### 3.1. Evaluation of Cutting Temperature Results

**Figure 3:**
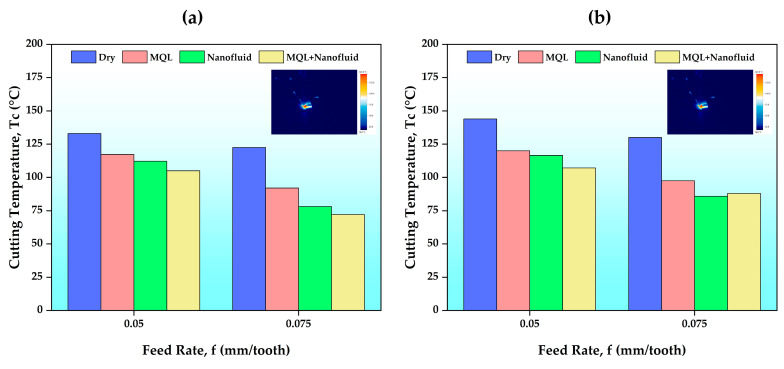
Cutting temperature results according to the feed rates of different C/L conditions: (**a**) Vc = 120 m/min, (**b**) Vc = 150 m/min.

It is crucial to control the cutting temperature to increase tool life, ensure the dimensional accuracy of the workpiece, and obtain the surface quality at the desired level. Cutting temperatures significantly impact machinability metrics such as surface quality and tool wear [33]. In this part of the evaluation, the effects of different C/L environments with varying cutting parameters on cutting temperature were examined, and the results are given in Figure 3. As a result of the experiments, it is observed that cutting temperatures decrease with increasing feed rate and that cutting temperatures increase with increasing cutting speed. The increase in feed rate caused the cutting tool–workpiece contact time to decrease. Thus, it can be said that the cutting temperature indirectly decreases as the time spent on friction decreases. In addition, as the feed rate increases, the quantity of chips produced per unit time increases, so the heat energy removed with the chips per unit time also increases. Thus, the cutting temperature decreases. This result is supported by literature studies [22,34]. As the cutting speed increased, the cutting temperature increased according to the literature [35,36] and Cook’s equation (Equation (1)).

T = Kv^m^, (1)

Here, T represents the measured temperature, K and m represent the constants of the workpiece material, and v represents the cutting speed. As expected, the highest cutting temperature (143.9 °C) was obtained in a dry C/L environment (Vc = 150 m/min and f = 0.05 mm/tooth). In the experiments carried out with the MQL system, the decrease in the cutting temperature can be seen from the results. However, there is an important point that stands out in these results. This critical part is the effect of C/L conditions on temperature. In particular, C/L fluids prepared with CNC nanopowder played an active role in reducing the cutting temperature. The lowest cutting temperature (72.2 °C) was achieved in the MQL + nanofluid C/L environment at a cutting speed of 120 m/min and a feed rate of 0.075 mm/tooth. In experiments performed with nanofluid alone, an average improvement in cutting temperature of 25% was achieved compared to dry, while an improvement of approximately 9% was achieved compared to MQL oil. When MQL oil–nanofluid was used together, the improvement in cutting temperature was found to be 30% compared to dry and 13% compared to MQL oil. This is attributed to the fact that CNC nanoparticles both improve the thermal properties of the base fluid in which the nanofluid is prepared, and combine excellent cooling and lubrication properties with the MQL oil under hybrid C/L conditions. Synthetic oil sprayed and pulverized by the MQL system helps reduce friction by creating a thin lubrication film between the cutting tool and the workpiece [37]. Additionally, a study emphasized that nanoparticles create a protective film and have repair, slip, and polishing effects between the workpiece and cutting tool, thus reducing the cutting temperature [24]. This can be explained by CNC-based nanofluids reducing the cutting temperature. In the literature, a machinability study using CNC-based nanofluids stated that different ratios of nanofluids played an effective role in reducing cutting temperatures [31]. Additionally, many studies reported that nanofluids prepared with different nanoparticles reduced the cutting temperature better than dry and MQL [38,39,40].

### 3.2. Evaluation of Surface Roughness Results

One of the most important factors indicating the machinability quality of a material is surface quality [41]. Surface quality significantly affects a material’s working principle, behavior, and functionality [42]. A quality surface can indirectly contribute to the strength and wear resistance of the material [43,44]. Arithmetic mean surface roughness (Ra) is one of the roughness criteria considered in determining surface quality [45]. Therefore, it is essential to evaluate surface roughness as a machinability metric. The expectation of improving the surface quality of such materials, which are especially preferred in engineering projects, continues.

**Figure 4:**
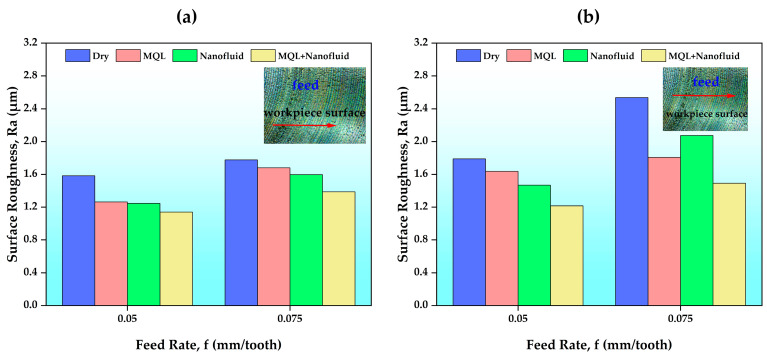
Surface roughness results according to the feed rates of different C/L conditions: (**a**) Vc = 120 m/min; (**b**) Vc = 150 m/min.

Figure 4 shows the surface roughness values for different C/L conditions at different feed rates. Figure 4a shows the measurement results at 120 m/min cutting speed, and Figure 4b shows the measurement results at 150 m/min. It was observed that when the cutting speed was increased, the surface quality decreased for all C/L conditions. With increasing cutting speed, the cutting temperature between the cutting tool and the workpiece increases [46]. Additionally, at high cutting speeds, more vibration and more wear may occur in the cutting tool. Thus, surface roughness values may increase. In the experimental results, the increase in feed rate (f) caused an increase in surface roughness values, as expected. This result confirms the numerical equation (Equation (2)) between f and surface roughness by showing real effects.

R_i_ = f^2^/(32r), (2)

Here, R_i_ is the ideal average surface roughness, f is the feed rate, and r represents the insert radius. When the test results were examined, the highest roughness value (2.534 µm) occurred due to the experiment performed in a dry environment. It should be noted that the highest cutting parameters were used in this experiment. Not using any C/L fluid during the cutting process causes excessive resistance between the cutting tool and w/p [47]. Thus, as expected, higher cutting temperatures lead to more tool wear and, ultimately, to a poor surface finish. It can be said that the MQL environment offers a higher quality surface than dry. This can be explained by the fact that the pulverized MQL oil particles penetrate the gaps on the workpiece surface. The most striking result is the increase in surface quality when using the hybrid C/L condition (CNC-based nanofluid + MQL oil. The best surface roughness value (1.139 µm) was obtained in the hybrid C/L environment (MQL oil + CNC nanofluid) with low cutting parameters. It was found that using CNC nanofluid increased the surface quality by an average of 17% and 1% compared to dry and MQL, respectively. Likewise, in the hybrid C/L environment, these rates were 26% and 13%, respectively. This situation can be interpreted as nanopowders acting as a repair on the workpiece surface in experiments performed with nanofluids. Moreover, surface quality improvement under hybrid C/L conditions can be attributed to the ball-bearing effect of nanoparticles combined with the lubricating effect of the MQL liquid. Studies in the literature support this situation [2,8].

### 3.3. Evaluation of Cutting Tool Wear

#### 3.3.1. Maximum Flank Wear

Tool wear is significantly affected by cutting parameters and C/L conditions. Tool wear is an important machinability feature because it substantially affects workpiece dimensions and surface quality [48]. Additionally, cutting tool life is a large part of machinability economics. The flank wear value, accepted as a criterion for evaluating the cutting tool life, can provide information, especially about the material’s machinability. According to the ISO 3685:1993 tool life test standard [49], for a tool to complete its life, it must reach an average flank wear value of 0.3 mm, a maximum flank wear value of 0.6 mm, or a severe tool failure criterion [40,50,51].

**Figure 5:**
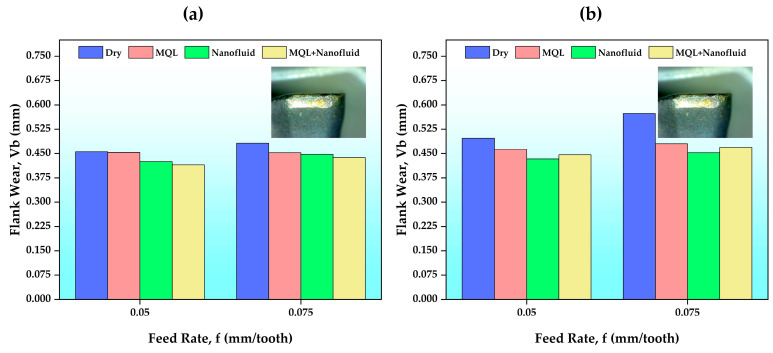
Flank wear results according to the feed rates of different C/L conditions: (**a**) Vc = 120 m/min; (**b**) Vc = 150 m/min.

The maximum flank wear (Vb_max_) results on the cutting tool after the experiments are presented in Figure 5. As expected, in the experiments carried out without using any cooling/lubricating fluid, the maximum flank wear value reached the highest value (0.573 mm). As the cutting speed and feed rate increased, flank wear values increased. However, the effect of the feed rate was not as much as that of the cutting speed. This situation is attributed in the literature to the increasing cutting temperature as the cutting speed increases [45]. The MQL environment creates a thin oil film between the cutting tool and w/p compared to dry, and this oil film reduces the friction force and friction coefficient. Thus, the tool wear value may be lower than that for dry. The experimental results performed in C/L environments where CNC nanofluids were used are interesting. The lowest flank wear value (0.415 mm) was obtained in the hybrid C/L environment at 120 m/min cutting speed and 0.05 mm/tooth feed rate. Although the hybrid C/L environment provided an average improvement in flank wear values of 12% compared to dry, this rate was approximately 5% compared to the MQL environment. These values were followed only by results obtained from experiments with CNC nanofluid. Nanofluids have significant effects in reducing the cutting temperature. Nanofluid also plays a role in reducing cutting forces by reducing the friction coefficient between the cutting tool and w/p. Thus, the force acting on the cutting tool decreases and can contribute to the decrease in wear value [12]. It was determined that the wear value of the hybrid C/L environment was 2% better than that of the CNC-based nanofluid. However, this advantage was seen at low cutting speeds. A higher cutting temperature is achieved at a higher cutting speed. Since only CNC-based nanofluid has good heat transfer properties, it provides better heat management and helps distribute heat. This can increase the wear resistance of the cutting tool. The fact that less cutting temperature was produced in the experiments using only nanofluid at a high cutting speed and high feed rate supports this opinion (see Figure 3). There may also be other reasons. In experiments performed in the hybrid C/L environment, more heat is produced at higher cutting parameters. Although CNC-based nanofluid helps reduce the burning effect, the chemical structure of the MQL fluid may change slightly with combustion. Residues resulting from changes in the chemical structure can cause both increased friction and damage to the cutting tool coating, and may thus have a negative impact on tool wear resistance.

#### 3.3.2. Flank Wear Mechanisms

Wear occurs on the cutting tool because of mechanisms such as mechanical, chemical, thermal, and fatigue mechanisms. Abrasion, adhesion, diffusion, and oxidation wear mechanisms, which are the most common in milling operations, can mostly be seen in cutting tools [3,52]. Cutting tool damage mechanisms must be known to determine and analyze tool life.

**Figure 6:**
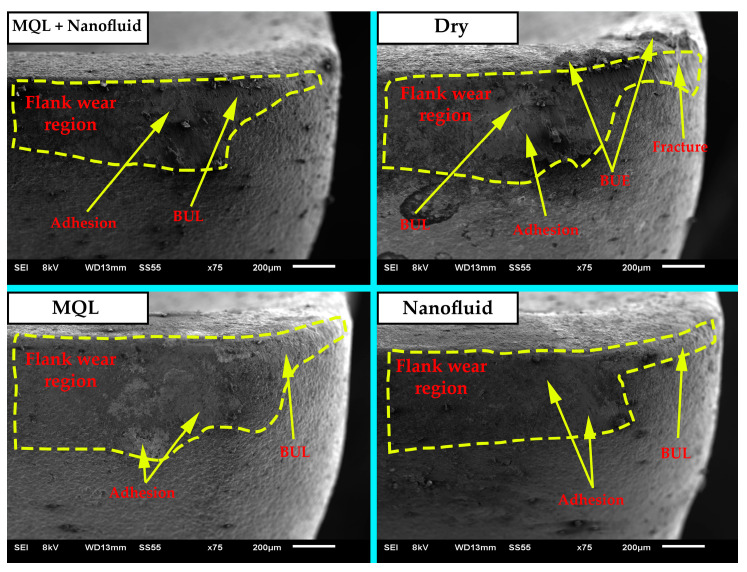
SEM photographs of wear mechanisms of cutting tools for different C/L environments.

**Figure 7:**
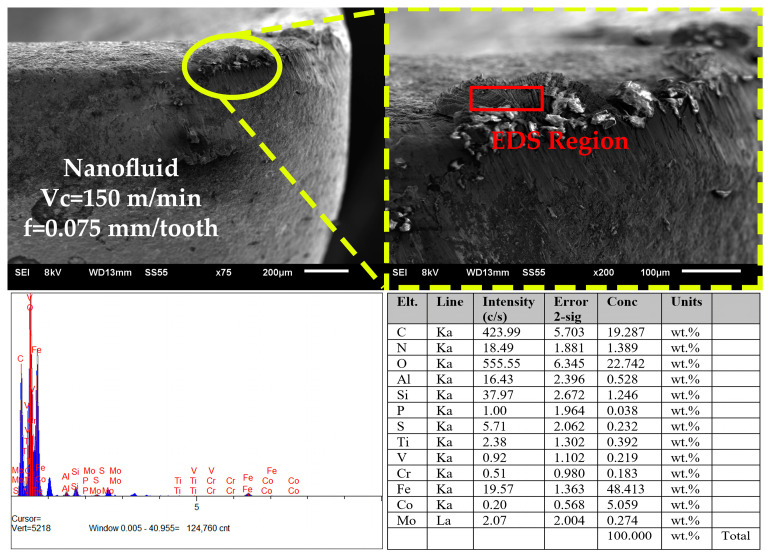
EDX analysis of the presence of BUE detected for the dry environment.

**Figure 8:**
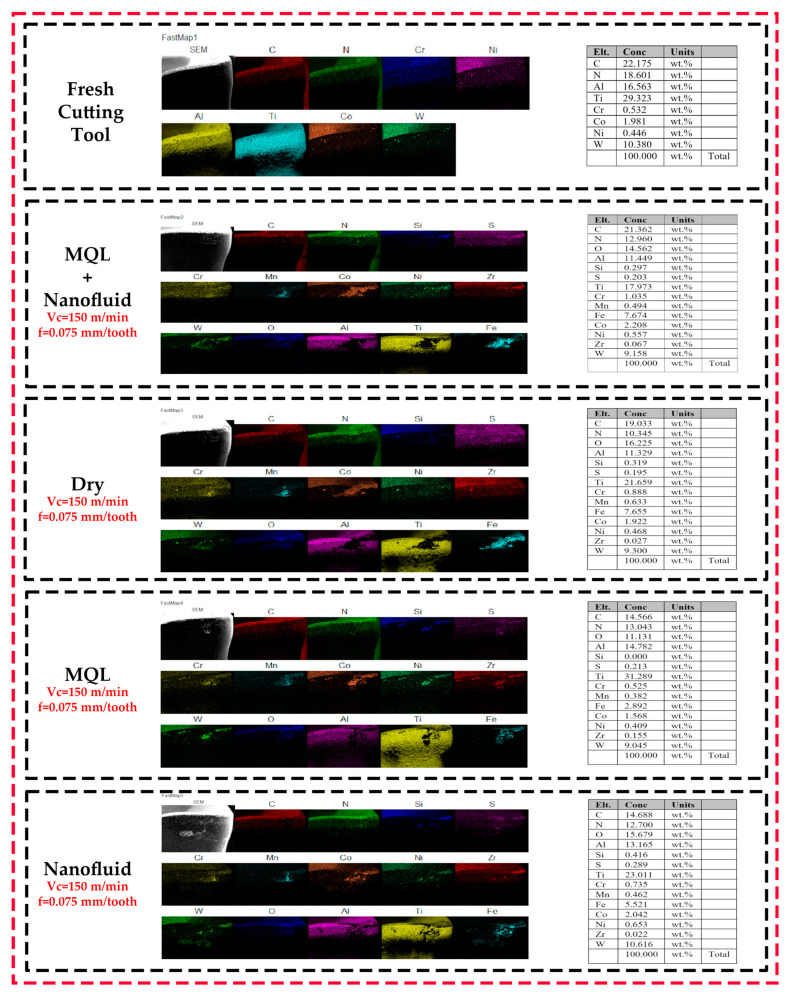
Elemental mapping analysis of cutting tools for different C/L environments.

Figure 6, Figure 7 and Figure 8 present the SEM, EDX, and elemental mapping analysis of the final state of the cutting tools after experiments conducted under the most difficult cutting conditions (Vc = 150 m/min and f = 0.075 mm/tooth) where maximum wear values are expected. As a result of the analysis, it was determined that the dominant wear mechanism was adhesion. As a result of this wear, built-up-layer (BUL) presence was found in the experiments carried out with cooling/lubrication fluids under the specified conditions. It is interesting that BUL assets continued with the introduction of cooling/lubricating fluids. BUL is recommended to reduce cutting parameters, and thus to reduce high cutting temperatures, which is the main reason for their existence. In addition, another situation caused by high cutting temperatures was chip debris, which was observed in the experiment performed in the MQL oil. It was observed that chip debris did not form in experiments using CNC-based nanofluids. This can be attributed to the fact that CNC-based nanofluids reduce the cutting temperature more effectively than MQL. Formation of cracks with abrasive wear was observed at high cutting parameters in a dry environment. The absence of any C/L environment can lead to high cutting forces and high cutting temperatures in the cutting process. Thus, thermal stresses may occur on the cutting tool and reduce tool resistance. It was determined that this situation disappeared with C/L fluids. Additionally, built-up-edge (BUE) and EDX analyses were performed in the experiments conducted in dry environments (Figure 7). The results obtained from the analyses made in the BUE region show the elements belonging to w/p. It was reported in a study that BUE formation may develop due to the effect of high cutting temperatures and mechanical loads. BUE formation may have a negative impact on surface quality by increasing cutting forces [25] (see Figure 4). BUE formation generally occurs with the use of MQL fluid and CNC-based nanofluids, resulting in a decrease in cutting temperatures by decreasing friction between the cutting tool and w/p. In this case, BUE formation can be prevented [40].

Figure 8 shows the presence of carbon (C), chromium (Cr), cobalt (Co), nickel (Ni), and tungsten (W) elements in the cutting tool material, as well as the Al-TiN coating for the fresh cutting tool. In addition, the presence of w/p elements and cutting tool materials in the experiments carried out at the specified cutting parameters proves that the primary wear mechanism is adhesion (see Table 1).

### 3.4. Evaluation of Energy Consumption Results

Minimizing energy consumption in an industry’s production process is critical. This process is inevitable for sustainable manufacturing. Some processes, such as reducing operating costs resulting from energy consumption, reducing environmental impact, and providing competitive advantage, make reducing energy consumption inevitable. In addition, it is necessary to measure the energy of the milling process to control the efficiency of such materials in the cutting process. One of the ways to control energy consumption in metal cutting processes is to use different C/L conditions [4]. While most of the energy consumption in industrial manufacturing occurs in machining, even the most minor improvements in machining will contribute significantly to the machining economy [3,53].

**Figure 9:**
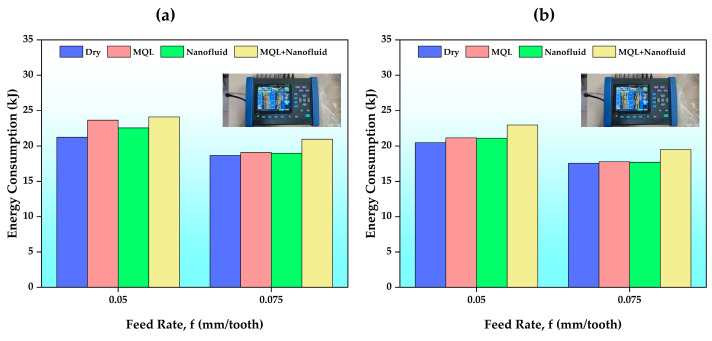
Energy consumption results according to the feed rates of different C/L conditions: (**a**) Vc = 120 m/min; (**b**) Vc = 150 m/min.

In this part of the study, the energy consumption effects of different C/L environments at different cutting parameters were analyzed (Figure 9). It should be noted here that the energy consumed by the MQL system was included in the total energy and the MQL system was not used in the dry environment. The energy consumed generally decreased with increasing cutting speed and feed rates. With increasing cutting speed and feed rate, the machine feed rate (table feed, Vf) increases, and the processing time decreases, which can reduce energy consumption. In addition, it can be said that increasing the cutting speed facilitates the deformation of the material and reduces energy consumption by reducing cutting forces [3]. The lowest energy consumption (17.57 kJ) was seen in the dry environment, where milling was performed without using the MQL system. The main reason for this situation is the lack of energy consumption of the MQL system. The highest energy consumption (24.1 kJ) was detected in the experiment using the hybrid cooling system. Despite the C/L advantage of MQL and nanofluid, the double MQL system consumed greater energy. Therefore, it was determined that the energy consumption in the MQL oil + CNC-based nanofluid (hybrid) experiments was higher than that in the experiments performed with CNC-based nanofluid. Although this decrease was more evident at low cutting parameters (4%), it can be said that it generally provides a 1% advantage. This situation can be explained by different aspects. Different C/L conditions affect the friction forces between the cutting tool and the workpiece. Increasing friction force can increase cutting forces, and the increase in cutting temperature confirms this situation (see Figure 3). In experiments performed with CNC nanofluid, nanoparticles can penetrate between the cutting tool and the workpiece, reducing the resulting friction forces. Thus, less energy consumption occurs compared to MQL since less cutting force is required [25]. On the other hand, since water-based nanofluids have lower viscosity values, they can penetrate the cutting tool–workpiece interface better [54]. Therefore, energy consumption can be reduced with a better lubrication process.

## 4. Conclusions

In this study, different C/L environments (CNC-based nanofluid and CNC nanofluid + MQL oil) were prepared using CNC nanopowders, and their effects on the machinability properties of DILLIMAX 690T material compared to other C/L media (dry and MQL oil) were investigated. In this context, the milling process was preferred to determine the machinability properties. The study’s main purpose was to investigate the applicability of C/L environments created with CNC nanopowders and to develop the machinability properties of the material used for w/p by deeply analyzing them. The following findings were obtained from this study:

- CNC-based nanofluid added to C/L environments significantly reduced the cutting temperatures obtained as a result of the experiments. The maximum cutting temperature obtained for the workpiece was reduced from 143.9 °C to 72.2 °C with appropriate cutting parameters and a hybrid (CNC nanofluid + MQL oil) C/L condition. Additionally, the hybrid C/L condition was approximately 5% more effective in reducing the cutting temperature than using CNC-based nanofluid.
- It was observed that the surface quality of the workpiece tended to decrease with the use of nanofluid. This convincingly proves that the hybrid C/L condition predominantly improves the surface quality of the material. It was determined that this hybrid C/L condition provides approximately 26, 13, and 10% improvements compared to dry, MQL oil, and CNC nanofluid, respectively. The hybrid C/L condition was proven to be a reliable fluid in improving surface quality.
- As a result of the cutting temperature, lower tool wear values were obtained at lower cutting temperatures. It can be said that the nanofluid C/L condition contributes as much as the hybrid C/L condition in reducing these values. They provided approximately 12% and 2% improvements in Vb_max_ values against dry and MQL oil C/L environments, respectively. The effect of cutting parameters confirms the literature studies.
- It was observed that the dominant wear mechanism occurring in cutting tools was adhesion. As a result, BUE and BUL mechanisms were seen in cutting tools. Although the use of C/L conditions prevented the formation of mechanisms such as chip debris, fracture, and BUE, it was observed that BUL formation continued. In addition, it was revealed that with appropriate C/L conditions, cutting parameters can be reduced slightly compared to high cutting parameters.
- Although dry machining seems to be the most advantageous for energy consumption, when the results obtained from other machinability metrics are considered, it is clear that dry processing negatively affects the machinability of the materials used. Since one MQL system is used for MQL oil and CNC nanofluid environments and two MQL systems are used for the hybrid C/L environment, it is natural that these C/L conditions have higher energy consumption than the dry environment. However, compared to using MQL oil, the energy saving was approximately 2% in experiments where only CNC-based nanofluid was used.
- As a result of the experiments, C/L environments produced with CNC nanopowder mixed with 0.5% by volume significantly improved material machinability in both single use and hybrid use. In general, it was observed that the hybrid C/L environment gave better results for machinability metrics compared to using CNC-based nanofluid.
- In future studies, different volume ratios can be tried to improve the machinability of similar or other metals. The studied approach can also be seen as a further alternative for turning, grinding, and drilling operations.

## Figures and Tables

**Table 1:** Chemical composition of Dillimax 690T material.

Elements	C	Si	Mn	P	S	Cr	Cu	Ni	Mo	B	Zr
Max %	0.2	0.6	1.6	0.02	0.01	0.8	0.3	2.0	0.7	0.005	0.015

**Table 2:** Design of experiments, 2 × 2 × 4 (full factorial design).

Parameters	Symbols	Unit	Levels			
			L1	L2	L3	L4
Cutting speed	Vc	m/min	120	150	-	-
Feed rate	f	mm/tooth	0.05	0.075	-	-
C/L conditions	C/L	-	Dry	MQL oil	CNC nanofluid	MQL oil + CNC nanofluid

**Table 3:** Technical properties of CNC nanopowders (from Nanografi).

Technical Properties	Value
Color	White/off white
Form	Spray dried powder
Average Particle Size	10–20 nm wide, 300–900 nm length
Density	1.49 g/cm^3^
Viscosity	>5 cP

## Data Availability

The data presented in this study are available on request from the corresponding author.

## References

[1] Marichamy S., Maniraj S., Thanigaivelan R., Kumaravel S.T., Vinoth Babu K., Mallesham P. (2021). Enhancement of material removal rate in EDM process using silicon carbide based strenx 900 steel. Mater. Today Proc..

[2] Rapoport L., Leshchinsky V., Lvovsky M., Nepomnyashchy O., Volovik Y., Tenne R. (2002). Mechanism of friction of fullerenes. Ind. Lubr. Tribol..

[3] Salur E. (2022). Understandings the tribological mechanism of Inconel 718 alloy machined under different cooling/lubrication conditions. Tribol. Int..

[4] Aslan A., Salur E., Kuntoğlu M. (2022). Evaluation of the Role of Dry and MQL Regimes on Machining and Sustainability Index of Strenx 900 Steel. Lubricants.

[5] Gitanjali V., Nithya P., Pandiarajan P., Dhruthi N., Vineeth Raj T., Subbiah R. (2021). Performance machinability through electrochemical grinding of strenx steel. Mater. Today Proc..

[6] Raguraman D., Sakthivel P., Paramasivam V., Girisha L., Krishnamoorthy S., Rahul Alex S., Subbiah R. (2022). Analyze the effect of abrasives in water jet Machining on strenx steel. Mater. Today Proc..

[7] Khanna N., Airao J., Kshitij G., Nirala C.K., Hegab H. (2023). Sustainability analysis of new hybrid cooling/lubrication strategies during machining Ti6Al4V and Inconel 718 alloys. Sustain. Mater. Technol..

[8] Makhesana M.A., Patel K.M., Krolczyk G.M., Danish M., Singla A.K., Khanna N. (2023). Influence of MoS2 and graphite-reinforced nanofluid-MQL on surface roughness, tool wear, cutting temperature and microhardness in machining of Inconel 625. CIRP J. Manuf. Sci. Technol..

[9] Gupta M.K., Korkmaz M.E., Sarıkaya M., Krolczyk G.M., Günay M., Wojciechowski S. (2022). Cutting forces and temperature measurements in cryogenic assisted turning of AA2024-T351 alloy: An experimentally validated simulation approach. Measurement.

[10] Kuntoglu M. (2022). Machining induced tribological investigations in sustainable milling of Hardox 500 steel: A new approach of measurement science. Measurement.

[11] Patel U.S., Rawal S.K., Arif A.F.M., Veldhuis S.C. (2020). Influence of secondary carbides on microstructure, wear mechanism, and tool performance for different cermet grades during high-speed dry finish turning of AISI 304 stainless steel. Wear.

[12] Ross N.S., Ganesh M., Ananth M.B.J., Kumar M., Rai R., Gupta M.K., Korkmaz M.E. (2023). Development and potential use of MWCNT suspended in vegetable oil as a cutting fluid in machining of Monel 400. J. Mol. Liq..

[13] Al-Shargabi M., Davoodi S., Wood D.A., Al-Musai A., Rukavishnikov V.S., Minaev K.M. (2022). Nanoparticle applications as beneficial oil and gas drilling fluid additives: A review. J. Mol. Liq..

[14] Şirin Ş. (2022). Investigation of the performance of cermet tools in the turning of Haynes 25 superalloy under gaseous N2 and hybrid nanofluid cutting environments. J. Manuf. Process..

[15] Gupta M.K., Demirsöz R., Korkmaz M.E., Ross N.S. (2022). Wear and Friction Mechanism of Stainless Steel 420 Under Various Lubrication Conditions: A Tribological Assessment with Ball on Flat Test. J. Tribol..

[16] Kamata Y., Obikawa T. (2007). High speed MQL finish-turning of Inconel 718 with different coated tools. J. Mater. Process. Technol..

[17] Ji M., Xu J., Chen M., Mansori M.E.I. (2020). Effects of Different Cooling Methods on the Specific Energy Consumption when Drilling CFRP/Ti6Al4V Stacks. Proc. Manufact..

[18] Tasdelen B., Wikblom T., Ekered S. (2008). Studies on minimum quantity lubrication (MQL) and air cooling at drilling. J. Mater. Process. Technol..

[19] Tosun N., Huseyinoglu M. (2010). Effect of MQL on Surface Roughness in Milling of AA7075-T6. Mater. Manufact. Process..

[20] Şap E., Usca Ü.A., Uzun M. (2022). Machining and optimization of reinforced copper composites using different cooling-lubrication conditions. J. Brazil. Soc. Mech. Sci. Eng..

[21] Şap S., Usca Ü.A., Uzun M., Kuntoğlu M., Salur E., Pimenov D.Y. (2022). Investigation of the Effects of Cooling and Lubricating Strategies on Tribological Characteristics in Machining of Hybrid Composites. Lubricants.

[22] Usca Ü.A., Uzun M., Şap S., Giasin K., Pimenov D.Y., Prakash C. (2022). Determination of machinability metrics of AISI 5140 steel for gear manufacturing using different cooling/lubrication conditions. J. Mater. Res. Technol..

[23] Babu M.N., Anandan V., Yıldırım Ç.V., Babu M.D., Sarıkaya M. (2022). Investigation of the characteristic properties of graphene-based nanofluid and its effect on the turning performance of Hastelloy C276 alloy. Wear.

[24] Yücel A., Yıldırım Ç.V., Sarıkaya M., Şirin Ş., Kıvak T., Gupta M.K., Tomaz Í.V. (2021). Influence of MoS2 based nanofluid-MQL on tribological and machining characteristics in turning of AA 2024 T3 aluminum alloy. J. Mater. Res. Technol..

[25] Şirin Ş., Kıvak T. (2021). Effects of hybrid nanofluids on machining performance in MQL-milling of Inconel X-750 superalloy. J. Manuf. Process..

[26] Lyu Z., Asadi A., Alarifi I.M., Ali V., Foong L.K. (2020). Thermal and Fluid Dynamics Performance of MWCNT-Water Nanofluid Based on Thermophysical Properties: An Experimental and Theoretical Study. Sci. Rep..

[27] Sun S., Sun S., Cao X., Sun R. (2016). The role of pretreatment in improving the enzymatic hydrolysis of lignocellulosic materials. Bioresour. Technol..

[28] Trache D., Hussin M.H., Haafiz M.K.M., Thakur V.K. (2017). Recent progress in cellulose nanocrystals: Sources and production. Nanoscale.

[29] Grishkewich N., Mohammed N., Tang J., Tam K.C. (2017). Recent advances in the application of cellulose nanocrystals. Curr. Opin. Colloid Interface Sci..

[30] Xie H., Du H., Yang X., Si C. (2018). Recent Strategies in Preparation of Cellulose Nanocrystals and Cellulose Nanofibrils Derived from Raw Cellulose Materials. Int. J. Polym. Sci..

[31] Şap S., Usca Ü.A., Tarih Y.S., Yar A., Kuntoğlu M., Gupta M.K. (2023). Novel Use of Cellulose Based Biodegradable Nano Crystals in the Machining of PPS Composites: An Approach Towards Green Machining. Int. J. Precis. Eng. Manuf. Technol..

[32] Ross N.S., Ganesh M., Srinivasan D., Gupta M.K., Korkmaz M.E., Krolczyk J.B. (2022). Role of sustainable cooling/lubrication conditions in improving the tribological and machining characteristics of Monel-400 alloy. Tribol. Int..

[33] Abukhshim N.A., Mativenga P.T., Sheikh M.A. (2006). Heat generation and temperature prediction in metal cutting: A review and implications for high speed machining. Int. J. Mach. Tools Manuf..

[34] Danish M., Gupta M.K., Rubaiee S., Ahmed A., Mahfouz A., Jamil M. (2021). Machinability investigations on CFRP composites: A comparison between sustainable cooling conditions. Int. J. Adv. Manuf. Technol..

[35] Bagherzadeh A., Budak E. (2018). Investigation of machinability in turning of difficult-to-cut materials using a new cryogenic cooling approach. Tribol. Int..

[36] Yıldırım Ç.V. (2020). Investigation of hard turning performance of eco-friendly cooling strategies: Cryogenic cooling and nanofluid based MQL. Tribol. Int..

[37] Duc T.M., Long T.T., Chien T.Q. (2019). Performance Evaluation of MQL Parameters Using Al_2_O_3_ and MoS_2_ Nanofluids in Hard Turning 90CrSi Steel. Lubricants.

[38] Moura R.R., da Silva M.B., Machado Á.R., Sales W.F. (2015). The effect of application of cutting fluid with solid lubricant in suspension during cutting of Ti-6Al-4V alloy. Wear.

[39] Zetty Akhtar A.M., Rahman M.M., Kadirgama K., Maleque M.A. (2020). Effect of TiO2 and Al2O3-ethylene glycol-based nanofluids on cutting temperature and surface roughness during turning process of AISI 1018. OP Conf. Ser. Mater. Sci. Eng..

[40] Anandan V., Naresh Babu M., Vetrivel Sezhian M., Yildirim C.V., Dinesh Babu M. (2021). Influence of graphene nanofluid on various environmental factors during turning of M42 steel. J. Manuf. Process..

[41] Binali R., Demirpolat H., Kuntoğlu M., Sağlam H. (2023). Machinability Investigations Based on Tool Wear, Surface Roughness, Cutting Temperature, Chip Morphology and Material Removal Rate during Dry and MQL-Assisted Milling of Nimax Mold Steel. Lubricants.

[42] Binali R., Demirpolat H., Kuntoğlu M., Salur E. (2023). Different Aspects of Machinability in Turning of AISI 304 Stainless Steel: A Sustainable Approach with MQL Technology. Metals.

[43] Şap S. (2023). Machining and Energy Aspect Assessment with Sustainable Cutting Fluid Strategies of Al–12Si Based Hybrid Composites. Int. J. Precis. Eng. Manuf. Technol..

[44] Şap E., Usca Ü.A., Gupta M.K., Kuntoğlu M., Sarıkaya M., Pimenov D.Y., Mia M. (2021). Parametric optimization for improving the machining process of cu/mo-sicp composites produced by powder metallurgy. Materials.

[45] Korkmaz M.E., Gupta M.K., Günay M., Boy M., Yaşar N., Demirsöz R., Ross K.N.S., Abbas Y. (2023). Comprehensive analysis of tool wear, surface roughness and chip morphology in sustainable turning of Inconel-601 alloy. J. Manuf. Process..

[46] Şap S. (2023). Understanding the Machinability and Energy Consumption of Al-Based Hybrid Composites under Sustainable Conditions. Lubricants.

[47] Thakur A., Manna A., Samir S. (2020). Experimental investigation of nanofluids in minimum quantity lubrication during turning of EN-24 steel. Proc. Inst. Mech. Eng. Part J J. Eng. Tribol..

[48] Demirpolat H., Binali R., Patange A.D., Pardeshi S.S., Gnanasekaran S. (2023). Comparison of Tool Wear, Surface Roughness, Cutting Forces, Tool Tip Temperature, and Chip Shape during Sustainable Turning of Bearing Steel. Materials.

[49] (1993). Tool Life Test Standard.

[50] Cappellini C., Abeni A. (2022). Development and implementation of crater and flank tool wear model for hard turning simulations. Int. J. Adv. Manuf. Technol..

[51] Gupta M.K., Mia M., Pruncu C.I., Kapłonek W., Nadolny K., Patra K., Mikolajczyk T., Pimenov D.Y., Sarikaya M., Sharma V.S. (2019). Parametric optimization and process capability analysis for machining of nickel-based superalloy. Int. J. Adv. Manuf. Technol..

[52] Aslan A. (2020). Optimization and analysis of process parameters for flank wear, cutting forces and vibration in turning of AISI 5140: A comprehensive study. Measurement.

[53] Mia M., Gupta M.K., Singh G., Królczyk G., Pimenov D.Y. (2018). An approach to cleaner production for machining hardened steel using different cooling-lubrication conditions. J. Clean. Prod..

[54] Talib N., Rahim E.A. (2018). Performance of modified jatropha oil in combination with hexagonal boron nitride particles as a bio-based lubricant for green machining. Tribol. Int..

